# The current status and challenges of bioethics education in undergraduate medical education in Karachi, Pakistan

**DOI:** 10.1186/s12909-024-05599-5

**Published:** 2024-07-09

**Authors:** Bushra Shirazi, Qamar Riaz, Aamir Mustafa Jafarey, Rashida Ahmed, Mohammad Shahid Shamim

**Affiliations:** 1 Department of Surgery and Centre of Biomedical Ethics and Culture, SIUT, Karachi, Pakistan; 2 Department for Educational Development, Aga Khan University, Karachi, Pakistan; 3 Centre of Biomedical Ethics and Culture, SIUT, Karachi, Pakistan; 4 Section of Histopathology, Department of Pathology and Laboratory Medicine, Aga Khan University, Karachi, Pakistan; 5 Aga Khan University, Karachi, Pakistan

**Keywords:** Bioethics, Medical education, Undergraduate medical curriculum, Challenges

## Abstract

**Background:**

The importance of including bioethics in the medical curricula has been recognized globally. Certain countries including Pakistan continue to lag behind although some developments have occurred recently.

**Objectives:**

The research aimed to provide a snapshot of bioethics education in undergraduate medical colleges in Karachi, Pakistan. The secondary objectives included identifying factors promoting or inhibiting integration of bioethics into the curriculum.

**Methods:**

A two-pronged strategy was used to collect data including a website review of medical colleges, in existence for more than ten years, recognized by the Pakistan Medical and Dental Council (PMDC), the regulating body for undergraduate medical education in Pakistan. The other arm employed in-depth interviews with medical educationists in colleges fulfilling inclusion criteria. Data from the website was analyzed and presented as frequencies. Qualitative data was analyzed using content analysis method which involved coding of transcripts, multiple readings and arriving at subthemes and themes iteratively.

**Results:**

Thirteen medical colleges were included for the website review, of which four were from public sector. Three medical colleges used the word “ethics” in their vision and mission statement and four had provided a detailed curriculum for ethics on their website.

Thematic framework included four broad themes: 1) Need for Bioethics Education, 2) Current Status of Bioethics Education 3) Challenges in integration of bioethics in medical curriculum and 4) Recommendations for integration of bioethics in the Curriculum. Participants were in agreement that bioethics was important in development of future physicians. Participants identified various challenges, foremost being shortage of trained faculty, lack of institutional buy-in and overcrowded curriculum.

**Conclusion:**

The study identified sporadic inclusion of bioethics in undergraduate medical curricula, left to the discretion of individual institutions. Since Karachi is a cosmopolitan city, the findings may reasonably reflect the situation in other parts of the country. While bioethics is recognized as an important field, it will continue to remain an orphan subject in the curricula unless the regulatory and accreditation bodies make it compulsory for institutions to include ethics in their curricula.

## Introduction

Medical education has increasingly paid attention to the inclusion of bioethics teaching in the curricula globally [1]. Certain countries however have lagged behind, for instance in the Asia–Pacific region such as Malaysia, and India [1, 2]. Pakistan, also a South Asian low-middle income country joins this group even though the accreditation body for undergraduate medical education the Pakistan Medical and Dental Council (PMDC) recommended introduction of bioethics education as a part of undergraduate medical curricula in 2002 [3]. While the guidelines explicitly stated that bioethics must be taught and assessed in medical programs, the introduction of bioethics education into the curriculum was left at the discretion of medical colleges. These institutions, both in the public and private sector were expected to develop their own strategies for the implementation of bioethics education.

During the past two decades, some medical colleges in the country included bioethics in their curricula. However, a report from 2011 demonstrated that bioethics was not taught as a formal part of the curricula, leaving education in this field optional rather than mandatory. Where taught, it was subsumed under different umbrellas such as forensic medicine or behavioral sciences [4]. Shaikh & Humayun also illustrate that where present, bioethics was shuffled between different disciplines including community medicine, behavioural sciences or jurisprudence [5].

Limited literature is available on the current status of bioethics in the undergraduate medical curriculum. Most have addressed the development of a bioethics theme in a single medical college, or assessed the need for bioethics education in Pakistan in general [6, 7]. The current status of bioethics education in the undergraduate medical curricula in Pakistan therefore remains largely obscure.

This study aims to provide a situation analysis of the status of bioethics in undergraduate medical education in Karachi, one of the main cosmopolitan cities of Pakistan. The paper, through a qualitative inquiry with key stakeholders in the field seeks to explore challenges associated with inclusion of bioethics in Pakistan along with methods to improve the current situation. The findings of this inquiry would be useful for the accrediting bodies to execute the inclusion of bioethics education in medical curricula across the country. It also holds lessons for other countries struggling with similar challenges of standardization and implementation.

## Methods

The study follows a cross sectional descriptive-exploratory study. It was conducted as part of a thesis to fulfill the requirements of Master’s in Health Professional Education in Karachi from November 2020 to April 2021 by the first author. Ethical approval was sought from the Ethical Review Committee of Aga Khan University (AKU), Karachi.

The study followed a two-pronged approach for data collection. One arm comprised of a website review of medical colleges and universities in Karachi to assess whether bioethics is included in the curriculum. A non-probability, purposive sampling was used to identify medical colleges and universities located in Karachi that had existed for more than ten years from the accrediting body (PMC/PMDC) website. The websites of these medical colleges were reviewed, and information related to the vision and mission statements, and the presence and placement of bioethics in the curriculum were recorded in a pre-designed data extraction form.

The second arm consisted of collecting data through semi-structured interviews with key stakeholders primarily medical educationists. The key stakeholder was considered as someone who was responsible for overseeing the curriculum and academic calendar. Emails were sent to academic leaders, such as the Dean and Principal of the 13 selected medical schools, requesting identification and subsequent permission to interview the key stakeholder in their institute. Reminder emails or telephonic messages were sent in case of non-response for two weeks. The identified focal persons were then contacted, and interviews were scheduled at their convenience.

Prior to initiating the interviews, informed consent was informed assuring the participants that their name or that of their institute would not be mentioned in any publication resulting from the research.

The first author (BS) conducted the interviews following an interview guideline developed specifically for the purpose of this research. Questions pertained to the status and the presence of bioethics in the institution, along with inquiry into the challenges of bioethics education specific to their context. The guideline also consisted of questions relevant to bioethics curriculum, teaching and assessment strategies used within each institute. Conducted in a mix of English and Urdu (the national language of Pakistan), the interviews lasted for 30 to 40 min. They were recorded with the participant’s permission. Data saturation was reached at approximately the 7th interview since similar codes and ideas were being expressed by the interviewees.

Interviews were transcribed verbatim by an office secretary and verified by the first author (BS) for accuracy by reading the transcripts while listening to the audio recordings. Subsequently, they were analyzed independently by two reviewers (AJ, MSS) who were chosen due to their familiarity with the field of bioethics education. The process of adding multiple reviewers allowed for reflexivity on part of the primary investigator and ensured rigor in data analysis. Codes were developed using inductive method of data analysis independently by all three reviewers using the content analysis method [8]. The coding focused only on manifest content due to the nature of the topic being studied. The reviewers and primary investigator met to arrive at a consensus for the final thematic framework which included subthemes grouped under broader themes.

The thematic framework was also reviewed by the supervisors for this thesis (QR) thus adding credibility to the findings of this research. Triangulation was achieved by ensuring data collection through two methods.

## Results

### Website review

Thirteen medical colleges fulfilled the inclusion criteria. Four out of the thirteen medical colleges were from the public sector, and the remaining were private. One medical college had no academic information on its website, while two were affiliated with a single university, hence sharing the website.

Eleven institutes stated their vision and mission statements, whereas two provided only the mission statement. The vision or mission statements of the institutes focused on matters of academic excellence, such as “centre of excellence,” “institution of distinction,” or “benchmarks for medical education.” Only three medical colleges used the word “ethics” in these statements, including “to produce ethical and dedicated professionals” and “medical education based on best ethical and evidence-based standards.”

Four out of the 13 medical colleges provided well-developed curricula on their website, while another four contained ethics-related objective integrated into different modules. The placement of ethics-related content was varied. At one medical college, medical ethics was placed in the dedicated slot for forensic medicine and identified 3–4 topics within the content. Other institutes placed the topics according to modules; for example, ethical concerns with reproductive health were placed in the respective clinical rotation.

One institute carried no information on bioethics on its website. None of the websites mentioned bioethics teaching or assessment as a separate entity or activity within the medical curriculum.

#### Qualitative arm

Fourteen key participants from 13 medical schools participated in the in-depth interviews. Ten were females. Five interviewees served as both clinicians and medical educationists within their institutes. Four participants possessed formal training in bioethics. Of these one also had a graduate degree in medical education. Detailed characteristics of participants are provided in Table 1.

**Table 1:** Participants’ characteristics

Variables	Domains	Frequency (n)
Sex	Male	4
	Female	10
Age	Above 50 years	10
	Less than 50 years	4
Qualification	Clinical postgraduate Diploma	5
	Diploma/ Master’s in Medical Education	2
	Diploma in Bioethics	4
Years of experience in medical education	More than 5 years	10
	Less than 5 years	4

Content analysis of the interviews generated four distinct themes and subthemes (see Table 2 for detailed thematic framework). The narrative however only provides the broad themes due to word count constraints encompassing elements that formed part of subthemes.

**Table 2:** Thematic framework and selective quotes from participants

Sr. no	Themes	Subthemes	Participants’ Quotes
1	Need for bioethics education	Shaping the individual	“Because none of us knows to behave professionally and ethically, we don’t know the official way of behaving with colleagues, patients, their relatives, or seniors. So there are boundaries that each profession has. And we are not made aware of these boundaries.” (P6)
		Cultural diversity	“*kaey baar kisi ka background different hota haey, moral values different hoti haen*…. [people coming from different backgrounds also have different moral values.].” (P8)
		Development of soft skills	“*aap aik doctor produce kar rahaey haen tou apko sirf aik full of knowledge robot nahi produce karna haey*… [You are producing a doctor not a robot providing only knowledge]”. (P11)
		Evolution of the educational system	“I actually did not know about pharma, because when I was doing my house job with… I went to a conference which was sponsored by a pharma company. And I had gifts and everything, and I had no clue. Absolutely no clue.” (P14) “Things need to be spelt out. It has to be now part of formal training. You cannot simply rely on seniors to teach you things.” (P7)
		Creation of Future Educators	“I believe the students of today who will graduate will find it easier to teach (bioethics) as they are already studying? the subject.”(P8)
2	Current Status of Bioethics Education	A neglected subject in Medical Colleges	“The institute [of medical education] is very new. Activities of the Centre for patient safety, professionalism and ethics is very, very new and under construction.” (P6) “patchy,” “varied,” “… done at the faculty’s initiative” (P3) “No perks (attached to the extra work)” (P5) “Nobody gets extra credit for it. We get praise for the extra work, but that’s it” (P14)
3	Challenges in the integration of Bioethics Education in Medical Curriculum	Shortage of Human resources	“I think faculty training is the major challenge…… we don’t have people to teach. And nowhere in Pakistan are there enough people to teach.” (P6)
		Negative Attitudes towards Bioethics Education	“Waste of time” (P11) and “did not realize the importance of teaching ethics. They don’t know what ethics is.” (P14). This lack of realization occurred mainly because individuals in more senior positions do not realize the importance of imparting ethics education. “[They say] we were trained in medicine without this subject.” (P11)
		Lack of Institutional buy-in	“There is resistance from these smaller colleges because one will have to hire faculty”. (P10) “[Bioethics] is not required by authorities.” “every department, every discipline, which has staff and faculty… it all costs money. Why would the institute spend on something that is not required by authorities?”(P3)
4	Recommendations for integration of bioethics in the curriculum	Faculty development	“course of a minimum of six months should be made mandatory [for all faculty members]” (P11)
		Standardization of Curriculum	“People should be identified, and everyone needs to come together.” (P10)
		Mandating Bioethics Education	“Regulatory bodies [should make] it a separate thing.”, “The only thing that can drive it is a regulation – a danda (stick). (P11)

##### Theme 1: Need for ethics education

Majority of study participants believed that bioethics requires a formal structure within the undergraduate medical curriculum for multiple reasons. While ethics may be considered “*tarbiat ka hissa* [part of grooming]” (P14), as stated by one participant, ethics education is necessary to “*polish*” (P14) the personality. It was pointed out that demonstrating ethical behaviour and correct bedside manner should not be taken for granted. Instead, it should be taught formally so that students realize the importance of demonstrating proper mannerism and integrity in their professional lives.

Participants believed that bioethics education allows appreciation of cultural diversity. Students from diverse backgrounds study in medical colleges and universities, and ethics education can help in developing tolerance and appreciation of different values. According to a participant, “*haar kisi ka background different hota haey, moral values different hoti haen*…. […people have different backgrounds with different moral values]” (P8).

Furthermore, participants were of the opinion that bioethics education allows for the development of integral communication skills necessary for fostering an excellent physician–patient relationship. It allows for the development of empathy and leads to lateral thinking. “*You are producing a doctor, not a robot providing only knowledge, you are producing a good human being, you are producing a good leader… bioethics is a part of this*” (P11).

Participants also believed that requirements of the educational system had changed dramatically in contemporary times. According to one participant, *“Things need to be spelt out. It has to be now part of formal training. You cannot simply rely on seniors to teach you things”* (P7). She provided the example that she was only able to understand the implications of interactions with the pharmaceutical industry after receiving formal education in bioethics, resulting a change in her clinical practice.

##### Theme 2: Current status of bioethics education

Interviews revealed that none of the studied medical colleges had a dedicated department or a unit responsible for bioethics education. Only one respondent in the medical college stated that her job description explicitly included overlooking and planning the curriculum for ethics education. Three institutes had mentioned bioethics education as a formal part of the curriculum, whereas, in one institute, bioethics education was done informally as a personal initiative by a faculty member with a postgraduate diploma in bioethics. In one private medical university, bioethics education existed for over two decades with a well-defined curriculum, identified pedagogies, outcomes, goals, and designated faculty members from various clinical and non-clinical departments. However, even this university does not have a separate bioethics department or unit.

Lack of standardization was seen across the institutes with the initiative largely person-driven rather than mandated by the institute. In most institutes, the teaching was *“patchy”* and *“varied”* primarily because it was *“done at the faculty’s initiative”* (P3).

Against this backdrop, faculty members who had initiated bioethics in their institutions believed that since teaching bioethics was not part of their job description, they did not receive any remuneration for it. According to one interviewee*, “the faculty go far and beyond”* in their line of duty, with “no perks” attached to the extra work they undertook (P5). As one participant stated laughingly*, “Nobody gets extra credit for it. We may get praise for the extra work, but that’s it”* (P14). Another related issue was that people are generally reluctant to pursue formal qualifications in bioethics since there is “*no [formal] career track*” (P3) in the field. However, faculty regarded their effort as *“giving something back to their institution”* which had supported them in their career development (P2).

##### Theme 3: Challenges in the integration of bioethics education in medical curriculum

All participants believed that finding the right person to teach bioethics was one of the biggest challenges, *“I think faculty training is the major challenge…we don’t have people to teach. And nowhere in Pakistan are there enough people to teach [bioethics]”* (P6). Even if interested faculty was available, they often did not know how to teach bioethics. As one stated, *“The issue is not just the content; the issue is also how to teach”* (P6).

Another interviewee, while discussing the shortage of human resources, explained the importance of all departments taking up components and playing their part, *“Each medical specialty has its ethical issues. Paediatrics will have its ethical issues as would the field of geriatrics…. So, the departments need to teach them, and there should be role models so students can learn through their actions/practice”* (P10, P12).

Lack of institutional buy-in was particularly prominent in more recently established colleges. Since the institutions did not realize the importance of bioethics, relegating it to a lower status within medical education and training, they did not want to invest in it. According to one participant in such a college, “*There is resistance from these smaller colleges because one will have to hire faculty”* (P10). This resistance naturally occurred because including this would mean that: “*we would need to make a slot in the timetable, will need to train faculty, will need to develop a curriculum and then implement it. It’s not an easy task to do…why would we want to have another thing burdened on us?*” (P14).

Institutions also demonstrated limited interest in establishing bioethics departments since *“It is not required by authorities (regulatory bodies).”* Participants believed that the push to develop such departments has failed because *“every department, every discipline, which has staff and faculty… it all costs money. Why would the institute spend on something that is not required by authorities (regulatory bodies)”* (P3).

These challenges were compounded since according to participants, many professionals considered teaching bioethics as a *“waste of time”* (P11) and *“did not realize the importance of teaching ethics*.” Participants believed that individuals occupying more senior positions did not realize the importance of imparting ethics education, since according to one participant, *“…they were not trained in it.”*

##### Theme 4: Recommendations for integration of bioethics in the curriculum

The interviews also explored potential solutions that the participants thought would help successfully integrate bioethics into the curriculum. Participants believed that one of the ways to do so would be to strengthen faculty by providing training resources. According to one participant, a minimum six month training should be made mandatory for all faculty, drawing a parallel to how formal qualification in medical education for all faculty was made mandatory.

Participants believed a standardized curriculum for bioethics would ensure better cohesion. As one participant stated, “*People should be identified (for curriculum development), and everyone needs to come together (for using it)*” (P8). Participants also recommended that institutions planning to initiate bioethics education should invite individuals from institutions where ethics has been taught for several years.

Study participants emphasized a top-down approach to address this issue, where a regulatory authority should mandate the inclusion of bioethics in the medical curriculum for accreditation since in their opinion, it was necessary to use *“danda [stick]”* (P11) for making ethics education a mandatory part of the curriculum for receiving accreditation from PMC (PMDC). Another added, *“The only thing that can drive it is a regulation. I am telling you if PMDC had not made a regulation that there should be a physiology department, anatomy department, biochemistry department in a medical college, then you would have medical colleges without these departments”* (P3).

## Discussion

The current work provides a situation analysis of bioethics education within the medical curriculum in specific undergraduate medical colleges/universities in the cosmopolitan city of Karachi. Efforts to document different aspects or components of bioethics education at specific institutes are present, but a holistic picture has largely been missing from the Pakistani literature. The paper therefore a valuable contribution to medical education literature with lessons for other countries who share similar issues.

Findings reflect that while bioethics education is gradually gaining prominence, it is largely driven by individuals with an interest in the field rather than structural adjustments. The variation in the curricula of different institutes demonstrates that the onus of responsibility is left to the institutes to develop its academics standards rather than a central authority mandating the inclusion of bioethics in the curricula. This has been mentioned from other literature in Pakistan [9].

While there is acknowledgment of the importance of bioethics education in the curricula to enhance ethical behavior, bioethics is seen as an “add-on” or considered “fluffy” and “soft.” In the current study, this was reflected in negative perceptions towards teaching of bioethics among many healthcare professionals, as told by study participants. This has also been evident from elsewhere among students who consider learning of ethics as lower priority compared to clinical knowledge and skills [2]. Since much of the curriculum is dedicated to enhancing scientific and clinical knowledge, the “soft skills” of professionalism and ethics tend to be ignored in an already over-crowded curriculum. This finding has been demonstrated from other parts of the world as well [1, 10]. This may very well be because of the viewpoint that while clinical skills are likely to generate clinical revenue, education in ethics with limited career opportunities is unlikely to be lucrative.

Another significant challenge reported by participants include the limited human resources available who have the required expertise to teach and implement bioethics. Lack of teaching capacity in the field of bioethics has been identified a major challenge within resource constrained settings including India [1, 11]. Medical students in Brazil, for instance, have also voiced these concerns with respect to qualified faculty available to handle the discussions generated in ethical discourse [12]. In Pakistan, the experience so far has been that medical educationists are typically hired to review the curricula and are tasked with the responsibility to include aspects of professionalism and bioethics. However, this creates practical issues. Medical educationists may not have background in teaching or assessing bioethics. For those handed this forced responsibility demonstrates a classic case of institutions “passing the buck” without providing the required infrastructure to the discipline by providing an appropriate human resource with subject knowledge. This is in contrast to the United States where medical ethics is required to be taught in medical colleges. However, even in the United States, there remains variation in the content included in the curriculum with instructors left to their own devices to organize the stated objectives of medical ethics courses [13].

The way human resource is utilized also complicates the picture of bioethics education in Pakistan. Since there is no remuneration to be expected for this field with individuals drawing their income from their primary fields, bioethics education will continue to remain neglected. One way of resolving this issue is to include bioethics within a larger umbrella of professional development, done in the context of two medical colleges in Hong Kong and Malaysia as reported by Ngan (2021) [2].

The top-down approach involved integrating ethics with humanities and law thereby addressing challenges of time and limited faculty available. This model may be improvised in Pakistan by adding a component the use of “dual appointments” and “value-added education” mentioned by the participants in which faculty are then remunerated for the additional work to increase extrinsic motivation.

Considering the multiple challenges identified by participants, it appears as if a top-down approach may be essential in formalizing bioethics education as part of the medical curriculum in Pakistan. Participants voiced that unless an initiative was undertaken on the part of the central regulatory authority mandating the establishment of bioethics departments, or regulations that make it necessary for institutes to include bioethics in their curriculum, bioethics would continue to remain a low priority. The case of India provides a relevant example. In 2019, the National Medical Commission of India introduced the Competency Based Curriculum in Medical Education for undergraduate medical students with a new module named Attitude, Ethics and Communication (AETCOM) across the country [14]. This change, instituted at the national level, has led medical colleges to adopt ethics and professionalism in a longitudinal fashion within their curricula. While long-term impact remains unknown at this point, a few studies have illustrated positive perceptions of this module among faculty and students alike [11, 15].

Another significant hindrance to this issue has been the uncertainty at the governmental level. In 2020, the federal government mandated the formation of Pakistan Medical Commission (PMC) to replace the existing regulatory structure, Pakistan Medical and Dental Council (PMDC) [16]. This was then reversed in 2022 [17]. Against this backdrop of structural adjustments and inconsistencies at the regulatory level, bioethics already an orphaned field is unlikely to receive its due importance. PMDC (as it is currently known) should ensure that bioethics should be a mandatory aspect of the curricula for institutions to receive the necessary accreditation. This will require lobbying from key stakeholders who are committed to upholding bioethics education in the country.

This research provides a situation analysis of bioethics education in the country albeit with a focus only on Karachi. However, this city can be considered a microcosm for the rest of the country, adequately capturing the current realities. The findings of this study also hold importance in terms of policy-making since it provides directions for institutes and accreditation bodies by identifying the gaps and challenges. It is hoped that the study will initiate a dialogue about inculcating bioethics in the curriculum and ignite interest in this field.

The study also has several limitations since it has looked at a sample of a selected group of people. Other stakeholders’ voices such as those of faculty in other fields, students and the administrative body can help provide a wider picture of the Pakistani context as far as bioethics education is concerned.

## Conclusions

The common challenges identified in this study across institutes are a lack of human resources and institutional commitment and more prominently, need for the implementation of strict policies from accrediting bodies. The study highlights a significant lack of bioethics education for medical students in Karachi, with varying levels of implementation across different institutions. While some institutions have been teaching bioethics for a long time and continuously improving their systems, the majority do not offer any bioethics education. It seems that there is a long path ahead before bioethics education in undergraduate medical education is well-established in the country.

## Data Availability

The data will be available from the corresponding author on reasonable request.
